# Multiple pathological mechanisms of Bin1 for tau aggregation and propagation

**DOI:** 10.1002/alz.090709

**Published:** 2025-01-03

**Authors:** Taisuke Tomita

**Affiliations:** ^1^ The University of Tokyo, Tokyo Japan

## Abstract

**Background:**

Recent genome‐wide association analysis has identified Bridging Integrator 1 (BIN1) as one of the genetic risk factors for late‐onset AD. In AD patients, single nucleotide polymorphisms in BIN1 correlate well with tau pathology, suggesting the involvement of BIN1 in the formation and propagation of tau accumulation pathology, but the molecular mechanism and point of action remain unclear.

**Method:**

To comprehensively clarify the effects of BIN1 on two pathological events, tau aggregation and propagation, we analyzed BIN1 knockout mice.

**Result:**

Using transgenic mice and seed injection model, we found that BIN1 knockout facilitated the cell‐autonomous tau accumulation in neurons but reduced the tau pathology induced by extra‐neuronal tau seeds. In the primary neuron‐glia co‐cultures, but not neuronal cultures, BIN1 knockout accelerated the degradation of the extracellular tau seeds. The present study suggested that BIN1 has different effects on tau metabolism in neurons and glial cells; the loss‐of‐function of BIN1 promotes the aggregation and accumulation in neurons, while BIN1 knockout enhanced the degradation mechanism of tau in glial cells.

**Conclusion:**

The present study implicates that BIN1 has different effects on tau metabolism in neurons and glial cells.

